# Identification of Online Health Information Using Large Pretrained Language Models: Mixed Methods Study

**DOI:** 10.2196/70733

**Published:** 2025-05-14

**Authors:** Dongmei Tan, Yi Huang, Ming Liu, Ziyu Li, Xiaoqian Wu, Cheng Huang

**Affiliations:** 1 College of Medical Informatics Chongqing Medical University Chongqing China; 2 Human Resources Department Army Medical Center Army Medical University (The Third Military Medical University) Chongqing China; 3 Department of Quality Management Army Medical Center Army Medical University (The Third Military Medical University) Chongqing China

**Keywords:** large pretrained language models, online health information, information identification, text similarity analysis, performance evaluation, ChatGPT, text generation, latent Dirichlet allocation, artificial intelligence

## Abstract

**Background:**

Online health information is widely available, but a substantial portion of it is inaccurate or misleading, including exaggerated, incomplete, or unverified claims. Such misinformation can significantly influence public health decisions and pose serious challenges to health care systems. With advances in artificial intelligence and natural language processing, pretrained large language models (LLMs) have shown promise in identifying and distinguishing misleading health information, although their effectiveness in this area remains underexplored.

**Objective:**

This study aimed to evaluate the performance of 4 mainstream LLMs (ChatGPT-3.5, ChatGPT-4, Ernie Bot, and iFLYTEK Spark) in the identification of online health information, providing empirical evidence for their practical application in this field.

**Methods:**

Web scraping was used to collect data from rumor-refuting websites, resulting in 2708 samples of online health information, including both true and false claims. The 4 LLMs’ application programming interfaces were used for authenticity verification, with expert results as benchmarks. Model performance was evaluated using semantic similarity, accuracy, recall, *F*_1_-score, content analysis, and credibility.

**Results:**

This study found that the 4 models performed well in identifying online health information. Among them, ChatGPT-4 achieved the highest accuracy at 87.27%, followed by Ernie Bot at 87.25%, iFLYTEK Spark at 87%, and ChatGPT-3.5 at 81.82%. Furthermore, text length and semantic similarity analysis showed that Ernie Bot had the highest similarity to expert texts, whereas ChatGPT-4 showed good overall consistency in its explanations. In addition, the credibility assessment results indicated that ChatGPT-4 provided the most reliable evaluations. Further analysis suggested that the highest misjudgment probabilities with respect to the LLMs occurred within the topics of *food and maternal-infant nutrition management* and *nutritional science and food controversies*. Overall, the research suggests that LLMs have potential in online health information identification; however, their understanding of certain specialized health topics may require further improvement.

**Conclusions:**

The results demonstrate that, while these models show potential in providing assistance, their performance varies significantly in terms of accuracy, semantic understanding, and cultural adaptability. The principal findings highlight the models’ ability to generate accessible and context-aware explanations; however, they fall short in areas requiring specialized medical knowledge or updated data, particularly for emerging health issues and context-sensitive scenarios. Significant discrepancies were observed in the models’ ability to distinguish scientifically verified knowledge from popular misconceptions and in their stability when processing complex linguistic and cultural contexts. These challenges reveal the importance of refining training methodologies to improve the models’ reliability and adaptability. Future research should focus on enhancing the models’ capability to manage nuanced health topics and diverse cultural and linguistic nuances, thereby facilitating their broader adoption as reliable tools for online health information identification.

## Introduction

### Background

The widespread global use of internet media has had a substantial influence on social interactions among individuals, communities, and societies [1]. An analysis by the advisory firm Kepios shows that active social media user identities had grown to 4.95 billion by the start of October 2023, equating to 61.4% of the world’s total population [2]. Recent years have witnessed a dramatic increase in consumer online health information seeking [3]. However, as access to information has become more convenient, the issue of false health information online has become increasingly prominent [4], posing significant risks to public health. Although there is no conclusive evidence that health misinformation spreads more broadly than scientific information, health misinformation reliably leads to misperceptions on health issues, which can have serious consequences for individuals’ health decisions [5]. In some cases, the spread of false health information has even led to uncontrollable consequences [6]. Furthermore, with the rise of fake news [7], internet users are facing growing challenges in discerning the authenticity of information [8]. False health information, often emotionally charged and highly shareable, spreads rapidly and has far-reaching negative effects. While public demand for understanding and accessing health information has increased, this has inadvertently contributed to the widespread dissemination of false health claims [9,10]. Therefore, scientifically evaluating online health information has become a critical issue that requires urgent attention.

The task of online information identification involves the automated classification and identification of various types and sources of information within vast amounts of online data with the goal of effectively identifying and using valuable information [11]. The identification of online health information is a subtask within this broader framework, primarily focused on classifying and discerning health-related content through textual analysis and feature extraction. This task heavily relies on natural language processing techniques for in-depth analysis, enabling the evaluation of the authenticity of online health information. This task not only requires models to have strong text classification capabilities but also necessitates their ability to identify key elements in online health information, uncover potential misleading content, and comprehensively assess the accuracy and reliability of the information. Compared to information in other fields such as entertainment, education, finance, and technology, online health information carries higher social sensitivity and risk due to its direct impact on public life and health [12]. Furthermore, as the internet becomes the primary source of health information for the public, the accuracy and reliability of online health information have significant implications for public health [13]. Therefore, this study evaluated the practical performance of various pretrained language models in online health information identification, aiming to better understand their effectiveness in addressing false health information and contributing to further research and practical applications.

On November 30, 2022, OpenAI launched ChatGPT. This large-scale pretrained language model quickly garnered widespread attention due to its advanced conversational abilities [14]. ChatGPT has shown impressive performance in areas such as search engine optimization and code development, and its extensive pretraining dataset also opens up possibilities for application in other specialized fields [15]. A review of previous studies reveals the evolution of text classification techniques from traditional machine learning algorithms to deep learning models [16,17] and, more recently, the emergence of pretrained models, which have become crucial tools for identifying online health information [18]. Models such as ChatGPT have also demonstrated relatively strong natural language processing and text generation capabilities in the medical field [19,20]. Reports indicate that ChatGPT, without specialized medical training, managed to pass the USMLE (US Medical Licensing Examination) with a qualified score, highlighting its potential in understanding and applying fundamental medical knowledge [21]. However, despite the advancements made by models such as ChatGPT across various domains, their effectiveness in the task of online health information identification still requires further research and evaluation.

Recently, research on the identification of false health information has received increasing attention. False health information has been defined as health-related information that lacks scientific evidence [22], which provides an important theoretical foundation for subsequent studies. It has been found that false news spreads faster, further, and to a larger audience on Twitter (now rebranded as X), with its wider dissemination primarily driven by the novelty of the information and emotional responses, especially in political contexts [23]. In addition, a social network analysis of Twitter data regarding COVID-19 and the 5G conspiracy theory revealed the characteristics of misinformation dissemination on social media platforms [24]. Another study tracked social media discourse during the COVID-19 pandemic and developed a public Twitter dataset on COVID-19 to study the patterns of misinformation spread [25]. It has also been noted that people’s insecurity about institutions led to many conspiracy theories and low-quality information during the pandemic, which deepened distrust in science and institutions and amplified the impact of these views and ideologies [26].

With the rapid advancement of technologies that produce artificial intelligence (AI)–generated content, particularly the widespread use of large language models (LLMs) such as ChatGPT, the focus has shifted from traditional machine learning to evaluating the authenticity and reliability of AI-generated content. One study pointed out the valuable role that LLMs could play in health care, particularly in supporting clinical work, research, and education, while also noting concerns about data privacy and dependence on these models [27]. Further analysis highlighted that ChatGPT-4 carries a higher risk of generating false information, with misinformation generation rates exceeding 80% [28]. This shift not only demonstrates the potential of AI technology in false health information identification but also introduces new risks and challenges [29].

### Objectives

**Figure 1:**
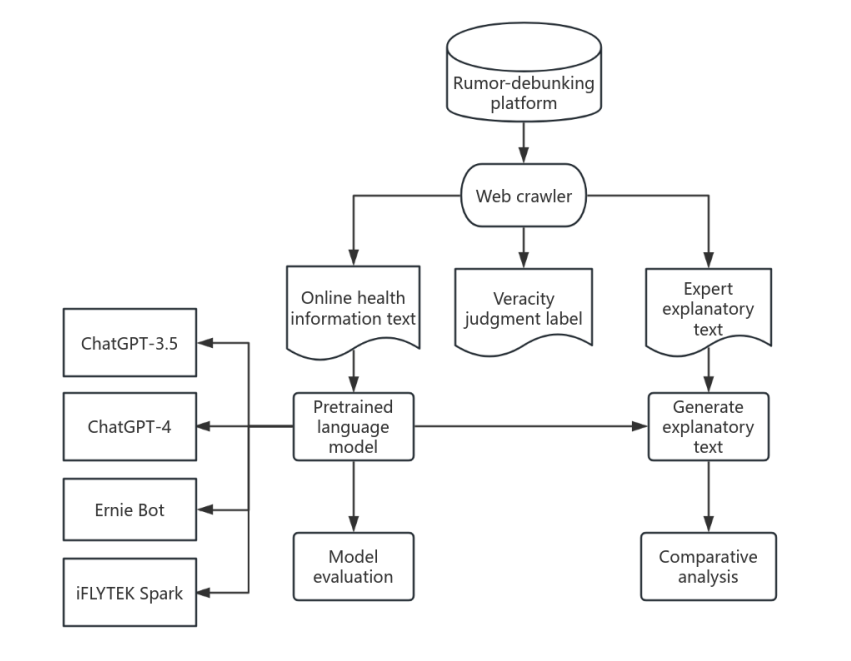
Framework flowchart.

In summary, the unique emotional characteristics of false health information and their significant impact on its dissemination highlight the urgent need for continuous optimization and improvement of current identification methods. Therefore, this study investigated the feasibility of applying 4 mainstream LLMs (ChatGPT-3.5 [OpenAI], ChatGPT-4 [OpenAI], Ernie Bot [Baidu], and iFLYTEK Spark) to identify online health information. This study was structured in 3 phases: data collection and preprocessing, model application and evaluation, and performance summary, as illustrated in Figure 1. In the first phase, data were gathered from domestic rumor-debunking platforms, followed by preprocessing to create a dataset containing 2708 pieces of online health information. The second phase focused on verifying this information using the 4 LLMs. The results from these models were then analyzed through data evaluation. Finally, the third phase involved analyzing the models’ identification performance and assessing their strengths, limitations, and practical application potential. Through empirical analysis, this study systematically evaluated the identification capabilities and performance of these models in real-world application scenarios. The findings of this study offer insights into the potential of LLMs for identifying false health information, which could inform future improvements in health misinformation detection methods.

## Methods

### Data Collection and Preprocessing

The dataset used in this study came from Tencent Fact Check, a Chinese platform that verifies misinformation by cross-referencing authoritative official sources and gathering debunking statements from various platforms. Verified sources include the World Health Organization, the National Medical Products Administration, and others. To ensure the clarity and completeness of the data sources, Multimedia Appendix 1 provides the website and specific verification sources. The platform conducts professional reviews to assess the authenticity of the information, ensuring the accuracy and reliability of the data labels. A Python-based (Python Software Foundation) web crawler collected a total of 3720 pieces of online health information, each annotated with authenticity labels and accompanied by expert or official explanations. During data preprocessing, the data underwent cleaning, label standardization, and format unification. The research team manually reviewed and removed redundant or semantically similar information, resulting in 2708 pieces of health information, which were then stored in a Microsoft Excel (Microsoft Corporation) file. Table 1 presents a portion of the online health information in the dataset, including expert assessments and explanatory texts. Of these 2708 pieces of health information, 680 (25.11%) were labeled as true, whereas 2028 (74.89%) were labeled as false. This distribution reflects the relatively high proportion of false information on the platform while also including a certain proportion of authentic information.

**Table 1:** Examples of the online health information in the dataset.

Information content	Expert assessment	Expert or institution explanatory text
Cancer cannot be prevented	False	“Some cancers can be prevented, while others do not have effective prevention methods. However, even for preventable cancers, taking certain measures does not guarantee a 100% certainty of not developing cancer; rather, it reduces the risk and probability of occurrence. Many cancers lack specific preventive measures, but for certain preventable cancers, such as lung cancer, there are numerous associated factors, both known and unknown. What we can do is to stay as far away as possible from known risk factors to minimize risk. Additionally, there are many cancers for which risk can be reduced through specific actions. For example, preventing hepatitis B virus infection can lower the risk of liver cancer; receiving the HPV vaccine and practicing safe hygiene can reduce the risk of cervical cancer; and changing poor dietary habits can decrease the risk of esophageal and gastric cancers, among others.”
Receiving the COVID-19 vaccine increases breast size	False	“Recently, some women who received the COVID-19 vaccine have noticed signs of breast enlargement. This phenomenon is actually a result of the body’s immune response, causing ‘swelling of the axillary lymph nodes,’ which is not unusual and is quite normal. This adverse reaction typically subsides on its own after a few weeks. The actual change occurs in the lymph nodes, not in the breast tissue, and receiving the COVID-19 vaccine does not have a breast enhancement effect. Lymph node swelling is not limited to the COVID-19 vaccine; it can be observed with many other vaccinations as well, so there is no need for excessive concern. However, lymph node swelling can also occur when the body is experiencing a bacterial or viral infection, in which case it is important to identify the specific cause and consider appropriate treatment.”
Over 90% of patients with hypertension have primary hypertension	True	“Hypertension is classified into two main categories: primary hypertension and secondary hypertension. The majority of hypertension cases in the population are primary hypertension, accounting for over 90%. Secondary hypertension constitutes a small portion, approximately 5-10%. The occurrence of primary hypertension is often due to multiple factors rather than a single cause. The increase in blood pressure is related to the patient’s living and working environment, dietary and exercise habits, and is typically the result of a combination of various influences. To prevent the rise in blood pressure, it is essential to address risk factors such as obesity, smoking, and alcohol consumption, adopt healthy dietary and exercise habits, and maintain a positive emotional state.”
Children cannot develop diabetes	False	“The occurrence of diabetes is the result of the interaction between genetic and environmental factors. The genes associated with type 1 diabetes come from both parents, and three environmental factors—climate, viral infections, and infant feeding practices—can also influence the development of type 1 diabetes. If one or both parents have type 1 diabetes, the likelihood of their children developing the condition is higher. Type 2 diabetes has a stronger familial hereditary tendency, which may be related to a family history of obesity and a high degree of similarity in unhealthy dietary and lifestyle habits within families. A healthy lifestyle can help delay or prevent the onset of type 2 diabetes.”

### Application Programming Interface Calls

In this study, a combination of application programming interface (API) calls and prompt design was used to obtain classification labels and explanatory texts generated by 4 LLMs for online health information, thereby facilitating subsequent evaluation. Specifically, the API calls served as the method of interaction with each model, whereas the prompt design ensured that these models produced consistent response formats. Table 2 summarizes the key steps involved along with the corresponding code functions, providing an overview of the entire process from environment setup to model interaction:

1. Data loading and preprocessing: health-related information was initially loaded from a Microsoft Excel file. Each record was individually processed and sent to the corresponding pretrained language models for verification to ensure data integrity and accuracy.
2. API calls: this study used specific functions to interact with various pretrained language models. The *open35_response(query)* and *open4_response(query)* functions were used to communicate with ChatGPT-3.5 and ChatGPT-4, respectively. The *spark35_response(query)* function enabled real-time interaction with Spark AI, whereas the *wenxin_response(query)* function was used to send queries to the Wenxin AI service. These functions transmitted user queries and received corresponding results to evaluate the authenticity of online health information, returning classification labels and their explanation texts.
3. Access token retrieval: to establish secure connections with the Wenxin API, the *get_access_token()* function was used to generate an access token. The function was authenticated using the API key and API secret, and the obtained token was used in subsequent API requests to ensure secure and accurate data transmission.
4. Overall process management: in the main function, the WebSocket connection for the Spark API was initialized, managing the overall query workflow. After retrieving data from the various models, the results were stored and formatted to facilitate the generation of a comprehensive view of online health information evaluations.

For effective evaluation, a uniform prompt structure was used across all models to ensure adherence to the same classification criteria and response format. The prompt was designed to request that the model to classify online health information as either true or false and provide a brief explanation for the classification decision. This approach was based on preliminary research findings, which indicated that most users interact with LLMs by posing simple, straightforward queries rather than engaging in more complex tasks such as fine-tuning or model pretraining. The prompt design aims to mimic typical user queries, making it both user-friendly and effective. Table 3 presents the unified prompt structure used for evaluating the authenticity of online health information, which includes the content, verdict, explanation, and an example. This structure allowed all LLMs to generate consistent classification labels and brief explanations, ensuring clarity and consistency in the evaluation results.

**Table 2:** Overview of application programming interface (API) calls and result processing workflow.

Step	Description	Code snippet or function
1	Set up environment	“os.environ” to set proxies
2	Define API functions	“open35_response,” “open4_response,” “wenxin_response,” and “spark35_response”
3	Read data from Microsoft Excel file	“read_xlsx(filename)”
4	Prepare data structure	Convert Microsoft Excel rows to a list of dictionaries
5	Initialize sample data	Add keys for model labels and reasons
6	Create prompt for AI^a^ models	Define a prompt template for the models
7	Read results from JSONL files	“read_jsonl(file_path)”
8	Extract and assign results	Use “re_patten(input_string)” to parse results
9	Process the results from each model	Loop through samples and assign labels and reasons
10	Output results	Print or save processed results

^a^AI: artificial intelligence.

**Table 3:** Prompt structure for evaluating the authenticity of online health information.

Step	Prompt component	Description
1	Information content	The health-related information to be judged (eg, “Drinking too much soda can cause leukemia”)
2	Verdict	The judgment result (eg, “False”)
3	Explanation	A brief explanation of why the judgment was made (eg, “Sweeteners are legal and safe food additives. The causes of leukemia are still not fully understood, and there is no scientific basis for the claim that sweeteners cause childhood leukemia. The claim that drinking soda leads to leukemia is false.”)
4	Example	The full prompt, including the information content, verdict, and explanation. Example: “Please judge the following content based on your knowledge, assess whether it is a rumor, return {true or false}, and provide a brief explanation. For example: {‘Information content’: ‘Drinking too much soda can cause leukemia,’ ‘Verdict’: ‘False,’ ‘Explanation’: ‘Sweeteners are legal and safe food additives. The causes of leukemia are still not fully understood, and there is no scientific basis for the claim that sweeteners cause childhood leukemia. The claim that drinking soda leads to leukemia is false.’}”
5	Prompt structure	The complete prompt structure for input into all models: Prompt=“Please judge the following content based on your knowledge, assess whether it is a rumor, return {true or false}, and provide a brief explanation. For example, see below: {‘Information content’: ‘Drinking too much soda can cause leukemia,’ ‘Verdict’: ‘False,’ ‘Explanation: ‘Sweeteners are legal and safe food additives. The causes of leukemia are still not fully understood, and there is no scientific basis for the claim that sweeteners cause childhood leukemia. The claim that drinking soda leads to leukemia is false.’} Please assess the following content {‘Information content’:”

### Performance Evaluation

#### Evaluation Metrics

The dataset contained 2708 health-related information samples. It covered a variety of online health information types, which was sufficient for evaluating model performance.

To construct a reasonable evaluation framework, this study incorporated precision, recall, and *F*_1_-score as the core evaluation metrics. The formulas for calculating these metrics are as follows.

Precision measures the proportion of true positive (TP) samples among all samples classified as *positive* by the model. The formula is as follows:



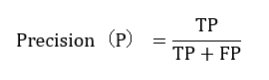



*TP* refers to the number of samples correctly classified as *positive* by the model, and *FP* refers to the number of *negative* samples incorrectly classified as *positive*.

Recall evaluates the proportion of actual positive samples that the model successfully retrieves. The formula is as follows:



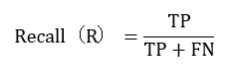



*FN* (false negatives) represents the number of positive samples that were incorrectly classified as *negative*.

The *F*_1_-score is the harmonic mean of precision and recall, providing a balance between the 2 metrics. The formula is as follows:



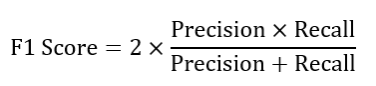



In processing 1.03% (28/2708) of the samples, for which the model outputs were labeled as *uncertain*, *undetermined*, *unknown*, *to be verified*, or *partly true partly false*, a conservative approach was applied. These outputs were considered model errors to ensure comprehensive coverage of potential true information. During the calculation, these samples were counted as false positives (FPs; if they were actually *negative*) or excluded from affecting TPs (if they were actually *positive*). In this study, although 7% (2/28) of the samples were labeled as *true* by experts, all 28 samples were treated as errors under the principle of *unknown equals wrong*, primarily impacting the FP calculation.

#### Model Evaluation and Analysis

In this study, we used 4 methods to evaluate and analyze the performance of the 4 LLMs: text length analysis, semantic similarity analysis, credibility assessment, and principal component analysis (PCA).

#### Text Length Analysis

Text length analysis is a method for statistically comparing the length of one or more texts to assess the characteristics of text output. By calculating the number of characters (average length, median, SD, minimum, and maximum) in the texts generated by medical experts and the 4 LLMs, this analysis provided a quantitative basis for the length features of the generated text. It revealed the diversity and complexity of text generation across different models. Although text length does not directly reflect the accuracy of the health information generated by the models, its distribution revealed the consistency and stability of the models in generating health information and highlighted the differences in text length characteristics across the models.

#### Semantic Similarity Analysis

Semantic textual similarity calculation is a key task in natural language processing that aims to assess the semantic consistency between 2 texts and quantify the degree of their similarity. This study used semantic similarity analysis to evaluate the semantic consistency between the texts generated by the models and the explanations provided by medical experts. This approach quantified the differences in semantic understanding between the LLM and expert explanations in the context of health information. This study used the jina-embeddings-v2-base-zh model developed by Jina AI. This model converts input text into fixed-length vectors (embeddings), ensuring that semantically similar texts are positioned closer together in the vector space. To quantify the similarity between model-generated text and expert explanations, feature data for both expert and model-generated texts were loaded from predefined data folders. The cosine similarity between expert features and model features was computed using the *F.cosine_similarity* function in PyTorch (Meta AI), reflecting the semantic consistency between the 2. The similarity scores for each model were calculated, and the mean, 75th percentile, and 25th percentile values were calculated. Finally, the similarity distributions were visualized using violin plots, with Matplotlib used to illustrate the similarity range and median for each model, providing a basis for assessing the degree of semantic matching between the model-generated content and expert explanations.

#### Credibility Assessment

To evaluate the ability of the 4 LLMs to assess the credibility of online health information, this study selected 52 online health information samples from various sources. The data sources included PubMed, Tencent Fact Check, Chunyu Doctor, Microblog, Bilibili, and REDnote, covering a wide range of information types and levels of evidence ranging from authoritative medical journals to online health information disseminated on social media. The selected samples encompassed both true and false health information and included various types of evidence, such as anecdotal evidence, observational studies, and randomized controlled trials (RCTs).

In modern medical practice, evidence-based medicine emphasizes the use of systematic evidence to assess the rationale behind clinical decision-making. To enhance the scientific rigor and precision of the evaluation, particularly in the assessment of online health information credibility, this study adopted the standard tool of evidence-based medicine—the Grading of Recommendations Assessment, Development, and Evaluation (GRADE) framework—as the evaluation tool. The GRADE framework is widely applied in the medical field and internationally recognized for its systematic approach to evaluating the quality of evidence and the strength of recommendations [30]. Moreover, the evidence quality is classified into 4 levels: very low, low, moderate, and high. High-quality evidence, often from well-conducted RCTs, suggests that further research is unlikely to change the results; moderate-quality evidence, typically from improved observational studies or RCTs with limitations, implies that more research could affect the findings; low-quality evidence, usually from flawed observational studies, suggests that further studies are likely to alter the conclusions; and very low-quality evidence, often from observational studies with numerous downgrading factors, indicates significant uncertainty in the results [31].

In previous related studies, methods such as semantic similarity have often been used, which focus solely on the surface similarity between texts. However, these methods fail to fully consider multidimensional factors such as the quality of the information, its source, and its clinical applicability. Therefore, the introduction of the GRADE framework for credibility assessment effectively addresses the limitations of semantic similarity–based evaluations, offering a more comprehensive and objective perspective. The specific steps are as follows. First, 2 independent researchers separately rated each sample according to the GRADE criteria, which included study design, risk of bias, consistency of results, directness and applicability, precision and CIs, and publication bias. Their ratings were then compared, and any discrepancies were identified. In cases in which disagreements occurred, the researchers engaged in discussions to resolve inconsistencies and reach a consensus on the final ratings. These finalized ratings were then used as the benchmark for evaluating the credibility of the online health information. Following this, the raw text of each online health information sample, along with relevant metadata (author information, source platform, and research type), was input into the 4 LLMs. When designing the prompts for the models, the GRADE rating criteria were explicitly provided, instructing the models to assess the credibility of the information based on its source and author qualifications and assign a credibility rating. Finally, interrater agreement between the ratings generated by the LLMs and those assigned by the research team was evaluated using the κ coefficient, and a heat map was generated to visualize the consistency of the credibility assessments across the models. This process provides a systematic approach for evaluating and comparing the performance of different models in assessing the credibility of online health information.

#### PCA Evaluation

PCA is a multivariate technique that reduces the dimensionality of data by transforming them into a set of new, mutually independent principal components, thereby extracting and visualizing key information in the data [32]. This study evaluated the similarity between the 4 LLMs and expert explanations by extracting text embeddings, reducing dimensionality using PCA, and performing visualization analysis. First, the API of each model encoded the health-related information explanation texts and extracted their respective feature embeddings. The same process encoded and extracted features from the experts’ explanation texts. Next, PCA was used to reduce the high-dimensional text features to 2 principal components for visualization in a 2D space. Specifically, after combining the experts’ text embeddings with those generated by each model, PCA was used to reduce the dimensionality of these feature representations, ultimately transforming the high-dimensional features into principal components in a 2D space. Finally, this study visualized the relative distributions of the experts’ and each model’s text embeddings in the same space. Feature distribution plots were generated for each model to compare the embeddings with those of the experts, offering an intuitive comparison of the semantic similarity between the model-generated explanations and the experts’ interpretations.

#### Latent Dirichlet Allocation Topic Modeling Analysis

To identify the latent topics within the 2708 online health information samples, the latent Dirichlet allocation (LDA) model was applied. LDA is an unsupervised learning algorithm used to infer latent topics in a set of documents, thereby revealing the deep structure of the text [33]. In this study, the LDA analysis was conducted as a post hoc analysis applied to the expert explanation texts after data collection and initial processing to ensure the extracted topics’ accuracy and representativeness and analyze the categories of misjudged instances.

The process was as follows:

1. Data preprocessing: during data preprocessing, the dataset consisting of 2708 online health information samples was cleaned by removing missing values and irrelevant data. Tokenization was then performed to break the text into words or phrases. To construct the feature vocabulary, the term frequency–inverse document frequency method was used to encode words in each document and extract the most representative features.
2. Topic number determination: the optimal number of topics was determined by evaluating multiple LDA models using perplexity and coherence scores. On the basis of these metrics, 7 topics were selected as the optimal balance between interpretability and model effectiveness.
3. Topic modeling: after determining 7 topics as optimal, the LDA model was applied to the dataset with this parameter setting. The model automatically identified the latent topics within the texts and produced the topic distribution for each document, along with the corresponding main topics. Analyzing the topic distributions uncovered latent semantic relationships between different online health information texts, providing valuable insights for subsequent accuracy assessment. The 7 topics extracted by the LDA model covered key areas of online health information, each exhibiting distinct semantic characteristics, providing a solid foundation for further analysis and model validation.

### Ethical Considerations

This study does not involve human participants or personal data. The data were collected from publicly available rumor-debunking platform and are anonymized. Since no interaction with individuals occurred, ethical review is not required under Article 32 of the Ethical Review Measures for Human Life Science and Medical Research (2023). Exemption has been granted by the College of Medical Informatics, Chongqing Medical University.

## Results

### Identification Results and Analysis

The 4 models demonstrated a high level of detail and fluency in generating explanatory texts, as illustrated in Multimedia Appendix 2. The first row of the table presents the results of the 4 LLMs in evaluating the following claim: “Diabetes is caused by eating too much sugar.” All models correctly identified this claim as false, consistent with the experts’ judgment. The second row shows the models’ evaluation results for the following claim: “Rice washing water can be used for beauty.” In this case, all models incorrectly classified the claim as true. Although the models made an incorrect judgment regarding this claim, the explanatory texts they generated still exhibited high fluency. In addition, Table 4 provides a comparison of the identification results for the 4 LLMs, showing the number of correct, incorrect, and uncertain classifications along with the evaluation metrics of accuracy, precision, recall, and *F*_1_-score. Of the 4 models, ChatGPT-4 achieved the best performance, with an accuracy of 87.27%, precision of 87.56%, and *F*_1_-score of 87.46%, indicating strong reliability. Ernie Bot and iFLYTEK Spark had accuracies of 87.25% and 87%, respectively, demonstrating a stable performance. In contrast, ChatGPT-3.5 had an accuracy of 81.82%, which was lower than those of the other models, suggesting that there is still considerable room for improvement in its ability to accurately identify online health information.

**Table 4:** Comparison of identification results of the large language models.

Model	Correct count	Incorrect count	Uncertain count	Accuracy (%)	Precision (%)	Recall (%)	*F*_1_-score (%)
Ernie Bot (Baidu)	2361	339	8	87.25	87.47	87.20	87.34
ChatGPT-4 (OpenAI)	2371	327	10	87.27	87.56	87.37	87.46
ChatGTP-3.5 (OpenAI)	2221	481	6	81.82	82.13	81.75	81.94
iFLYTEK Spark	2353	351	4	87.00	87.02	86.89	86.95

**Figure 2:**
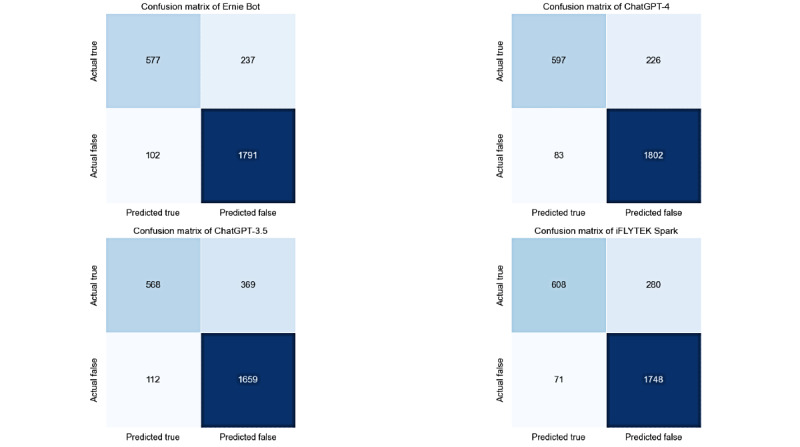
Confusion matrices.

Analysis of model performance showed that all 4 models performed well in distinguishing between true and false information. The TP and true negative values for each model were relatively high, whereas the FP and false negative values were relatively low, indicating that the models have a certain degree of reliability in filtering incorrect information and identifying accurate online health information. Figure 2 presents the confusion matrices for the 4 models, where the ratio of true negatives to FPs reflects the models’ ability to filter out incorrect information, whereas the ratio of TPs to false negatives reveals their performance in identifying true health information.

### Text Analysis

#### Text Length Analysis

**Figure 3:**
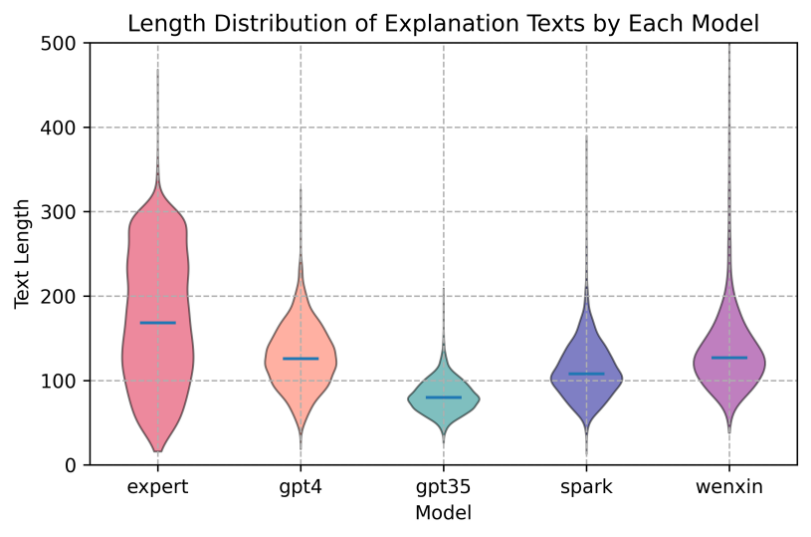
Text length distribution of the explanations from experts, ChatGPT-4, ChatGPT-3.5, iFLYTEK Spark (Spark), and Ernie Bot (Wenxin).

Figure 3 presents the length similarity violin plot of explanation texts generated by the experts, ChatGPT-4, ChatGPT-3.5, iFLYTEK Spark, and Ernie Bot. According to the plot, the text lengths of ChatGPT-4 and Ernie Bot were closest to those of the experts’ explanation texts, showing more consistent text generation characteristics. In contrast, ChatGPT-3.5 generated the shortest texts, with its text length distribution significantly lower than those of the other models, indicating greater variability. Table 5 summarizes and compares the statistical analysis of text lengths for the experts and the 4 LLMs, providing detailed information on mean, median, SD, and coefficient of variation. According to the statistical analysis in Table 5, the mean text length was 172.83 (SD 75.5; with a coefficient of variation of 43.68%) for the experts, 128.70 (SD 37.52; with a coefficient of variation of 29.16%) for ChatGPT-4, a mean of 81.73 (SD 19.15; with a coefficient of variation of 23.43%) for ChatGPT-3.5, a mean of 112.97 (SD 33.72; with a coefficient of variation of 29.85%) for iFLYTEK Spark, and 136.05 (SD 53.05; with a coefficient of variation of 38.99%) for Ernie Bot. The statistical results indicate that Ernie Bot’s text length was most similar to that of the experts. Although its coefficient of variation was marginally higher than those of ChatGPT-4 and iFLYTEK Spark, it still demonstrated notable stability and consistency. On the other hand, the text length generated by iFLYTEK Spark was relatively short with less variability. In contrast, ChatGPT-4’s text length and degree of dispersion were closer to those of the medical experts, highlighting its relative strength in generating explanation texts.

**Table 5:** Statistical analysis results for text length.

Model	Length, mean (SD)	Length, median (IQR)	Length range	Coefficient of variation (%)
Experts	172.83 (75.5)	170 (111.75-237)	18-355	43.68
ChatGPT-4 (OpenAI)	128.7 (37.52)	127 (102-153)	19-326	29.16
ChatGPT-3.5 (OpenAI)	81.73 (19.15)	80 (68-94)	26-275	23.43
Ernie Bot (Baidu)	136.05 (53.05)	127 (105-153)	38-797	38.99
iFLYTEK Spark	112.97 (33.72)	108 (90-132)	13-390	29.85

#### Semantic Similarity Analysis

**Figure 4:**
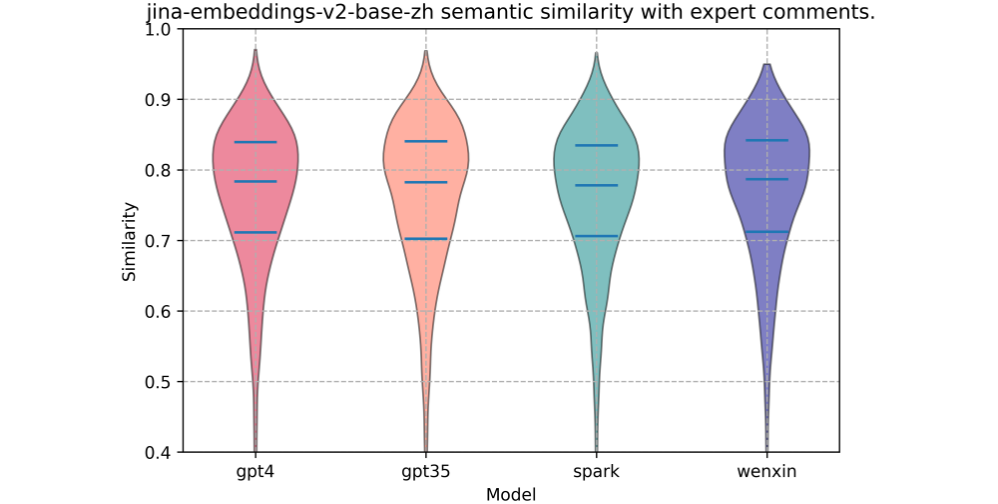
Distribution of semantic similarity between texts explained by ChatGPT-4, ChatGPT-3.5, iFLYTEK Spark (Spark), Ernie Bot (Wenxin), and experts.

Through semantic similarity analysis, we found that the texts generated by the 4 pretrained language models exhibited relatively high semantic similarity to the experts’ texts. Ernie Bot showed an advantage, demonstrating its ability to understand and generate content with semantics closely aligned with the experts’ explanations. Figure 4 presents the violin plot of semantic similarity, illustrating the distribution of similarity scores between the model-generated texts and the experts’ explanations. Table 6 further presents the detailed statistical data on the semantic similarity between the model-generated texts and the experts’ explanations. The table includes statistical metrics for each model, such as mean similarity, SD, coefficient of variation, 25th and 75th percentile similarities, maximum, minimum, and median similarity. The mean semantic similarity scores for ChatGPT-4, ChatGPT-3.5, Ernie Bot, and iFLYTEK Spark were 0.7622 (SD 0.0652), 0.7592 (SD 0.07), 0.7656 (SD 0.0657), and 0.7588 (SD 0.0657), respectively. The corresponding median scores were similarly high: 0.7836 (IQR 0.7116-0.8394) for ChatGPT-4, a median of 0.7825 (IQR 0.7027-0.8405) for ChatGPT-3.5, a median of 0.7869 (IQR 0.7124-0.8419) for Ernie Bot, and 0.7783 (IQR 0.7062-0.8349) for iFLYTEK Spark. These findings suggest that, in most cases, the models are effective in capturing the semantic nuances of online health information and generating texts that are comparable to expert explanations. Notably, Ernie Bot achieved the highest mean and median similarity scores, suggesting that its generated texts were the closest to the experts’ explanations.

**Table 6:** Statistical analysis results for semantic similarity.

Model	Similarity, mean (SD)	Coefficient of variation	Similarity, range	Similarity, median (IQR)
ChatGPT-4 (OpenAI)	0.7622 (0.0652)	0.0856	0.0501-0.9705	0.7836 (0.7116-0.8394)
ChatGPT-3.5 (OpenAI)	0.7592 (0.07)	0.0922	0.009-0.9686	0.7825 (0.7027-0.8405)
Ernie Bot (Baidu)	0.7656 (0.0657)	0.0859	0.0874-0.9498	0.7869 (0.7124-0.8419)
iFLYTEK Spark	0.7588 (0.0657)	0.0866	0.0369-0.9658	0.7783 (0.7062-0.8349)

**Figure 5:**
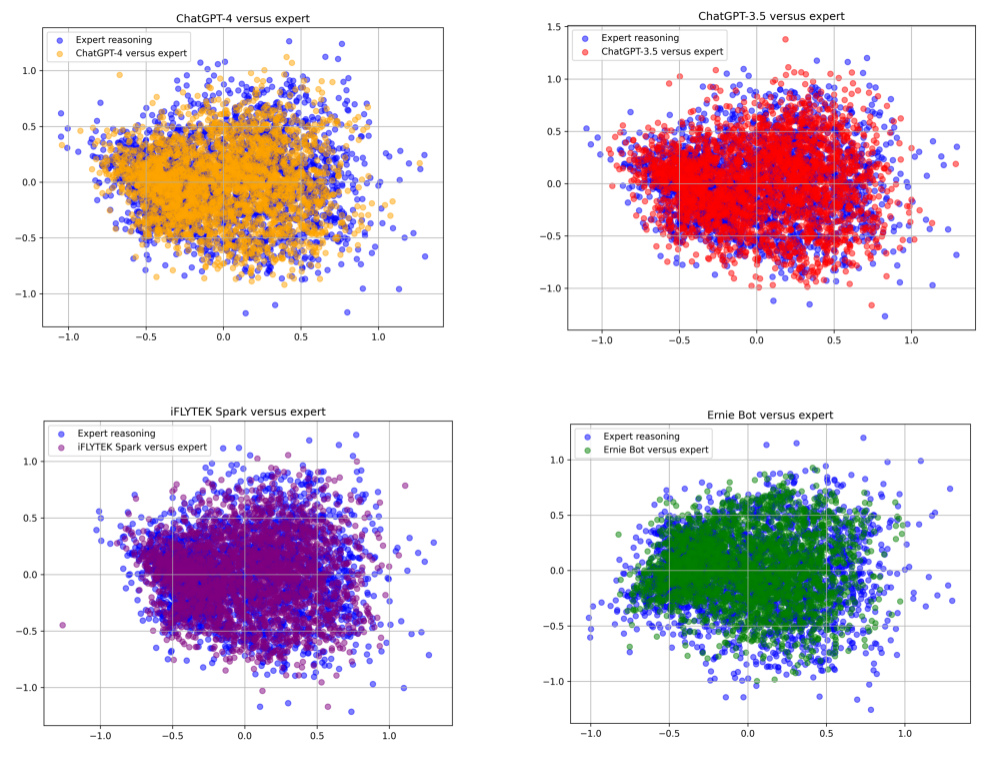
Principal component analysis dimensionality reduction visualization of texts generated by pretrained language models and of expert explanations.

In addition, PCA was used to reduce the dimensionality of the features of the texts generated by each model and the experts’ explanations and visualize them in a 2D space. Figure 5 illustrates that the projection points of the explanation texts generated by each model, when compared to the experts’ explanation text, generally cluster in adjacent areas. Some of these points are very close to the experts’ projection points, indicating a high degree of similarity in semantic content between the model-generated texts and the experts’ explanations. However, there are a few projection points that are quite far from the experts’ projection points, indicating that some models’ generated texts still show significant differences from the experts’ explanations.

In summary, the 4 models demonstrated relatively high semantic similarity to the experts’ explanation texts and exhibited consistency, but there were still noticeable differences in understanding certain online health information.

#### Text Content Analysis

To further evaluate the performance of the LLMs in understanding online health information, representative samples were selected for content analysis and summarization across the different models. As shown in Multimedia Appendices 3 and 4, the models exhibited varying performances in semantic similarity analysis. Multimedia Appendix 3 presents examples in which the models achieved higher semantic similarity to expert explanations, whereas Multimedia Appendix 4 highlights examples with lower semantic similarity. These variations reveal differences in the models’ ability to match semantics and in the depth of understanding when processing online health information.

The data from Multimedia Appendices 3 and 4 show that, for some online health information, the texts generated by the models exhibited high semantic consistency with expert recommendations. For example, regarding the following question—“Are lumps and nodules always cancer?”—the response generated by ChatGPT-3.5 showed a high degree of semantic similarity to the experts’ explanation, with a similarity score of 0.9686. This indicates that, in certain contexts, pretrained language models can accurately understand and restate complex health knowledge. However, when addressing the false information that “Vitamin C can boost immunity and prevent colds,” ChatGPT-3.5’s generated text significantly diverged from the experts’ opinion, with a semantic similarity score of 0.3684. Specifically, the experts’ explanation highlighted the limitations of vitamin C in preventing colds and offered a comprehensive set of prevention strategies, including regular exercise, a regular sleep schedule, handwashing, and maintaining a clean environment. In contrast, ChatGPT-3.5 mentioned that vitamin C may help enhance immunity but failed to adequately address its limitations and lacked detailed prevention advice. This explanation may result in users misunderstanding the information. This comparison underscores the limitations of pretrained language models when processing complex online health information and highlights the importance of integrating expert explanations and judgment to ensure the accuracy and reliability of the information.

iFLYTEK Spark demonstrated a semantic similarity of 0.9658 with the experts’ explanation when addressing the following misleading claim: “Cosmetic allergy causes vitiligo.” However, when responding to the following question—“Does applying facial masks provide hydration for the skin?”—the generated text showed lower similarity to the experts’ opinion. The expert text explained that the skin’s ability to absorb moisture is limited and that the main function of a face mask is to promote the absorption of active ingredients and provide physical cooling, not to hydrate. The expert text also emphasized the importance of moisturizing and warned that frequent wet applications could damage the skin barrier. In contrast, iFLYTEK Spark focused on the moisturizing effects of face masks, mentioning ingredients such as hyaluronic acid and glycerin, but it lacked a detailed scientific explanation and risk warnings, which could lead to misunderstandings. This highlights the model’s limitations in addressing certain health topics, suggesting a need for further training to improve its explanatory capabilities.

**Figure 6:**
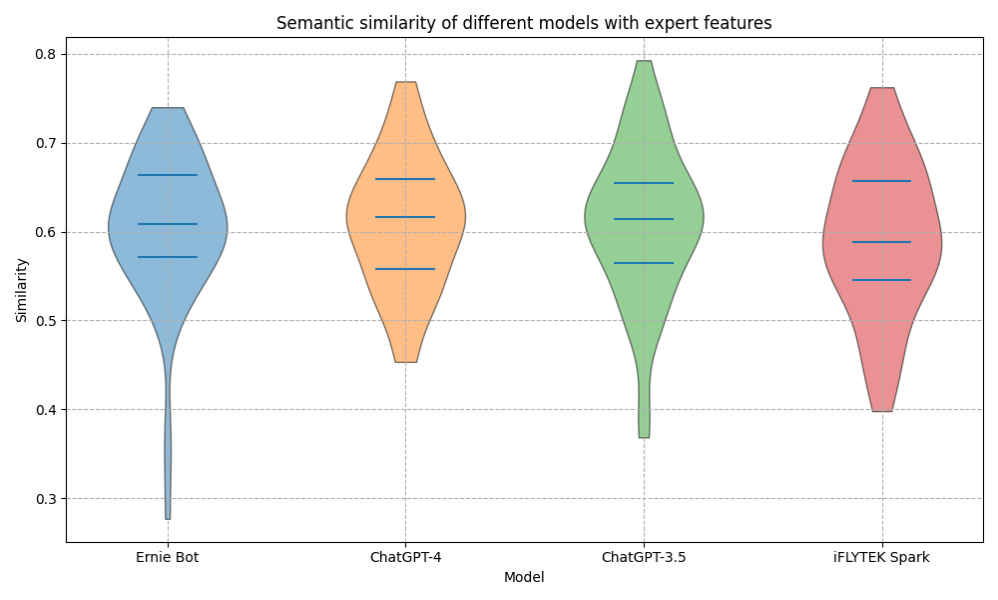
Comparison of semantic similarity between model explanations and expert explanations for misclassified online health information.

In Figure 6, the semantic similarity between the texts generated by the 4 LLMs after misjudging online health information and the experts’ explanations is further illustrated using a violin plot, highlighting the differences in performance among these models. This figure clearly shows that the semantic similarity of the model-generated explanation texts after misjudging online health information was lower than that of the overall sample. This indicates that, when the model fails to accurately process online health information, the semantic distance between the generated texts and the experts’ explanations increases. Moreover, the figure indicates that ChatGPT-4 performed better than the other 3 models in maintaining the minimum semantic similarity. Even in cases of misjudgment, its generated explanation texts still maintained a relatively high degree of semantic similarity. This suggests that ChatGPT-4 retains relatively strong language comprehension and text generation abilities when handling complex or controversial online health information.

#### Credibility Assessment

**Figure 7:**
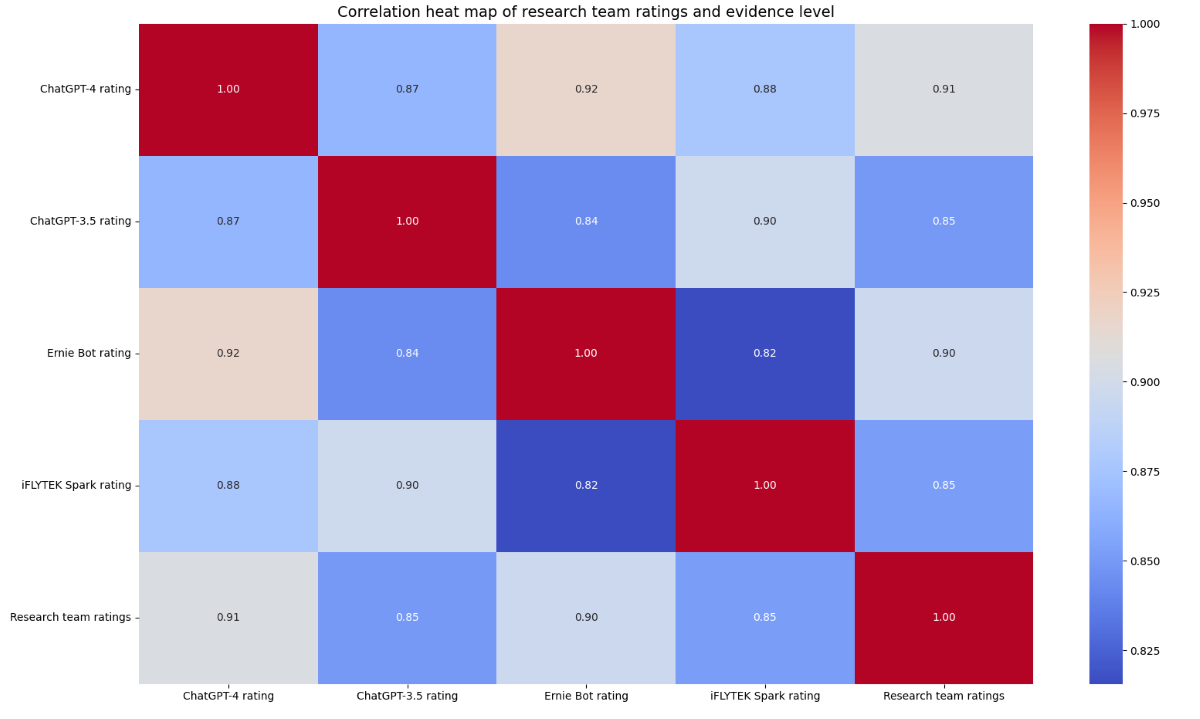
Correlation heat map of research team ratings and large language model (LLM) ratings.

To quantify the consistency between the credibility assessment results of the 4 LLMs and the ratings provided by the research team members, we calculated the κ coefficient and generated a heat map. The κ coefficient measures the level of agreement between the models’ credibility evaluations and the research team members’ ratings. Table 7 presents the κ coefficients for the 4 LLMs in relation to the research team members’ ratings. Specifically, the κ coefficient for ChatGPT-4 was 0.58, indicating a moderate level of agreement. Ernie Bot achieved a κ coefficient of 0.52, reflecting a certain degree of consistency. ChatGPT-3.5 had a κ coefficient of 0.42, suggesting a lower level of agreement, whereas iFLYTEK Spark exhibited the lowest agreement, with a κ coefficient of 0.31. In addition, the heat map (Figure 7) further visualizes the performance of the 4 LLMs in the credibility assessment. The results indicate that the ratings generated by ChatGPT-4 were the most aligned with those of the research team members, followed by the ratings generated by Ernie Bot.

**Table 7:** κ coefficients between the models and the research team members’ ratings.

Model	κ value
ChatGPT-4 (OpenAI)	0.5833
ChatGPT-3.5 (OpenAI)	0.4198
Ernie Bot (Baidu)	0.5201
iFLYTEK Spark	0.3131

#### Misjudgment Text Feature Analysis

To analyze the expert-explained texts for the 2708 online health information samples, we used LDA topic modeling. Table 8, based on the results of LDA topic analysis, presents the sample size and proportion of each topic in the total dataset, along with the number and proportion of consistently misjudged online health information across the 4 LLMs within each topic. To be more precise, experiments determined that the optimal number of topics was 7. These topics were *food and maternal-infant nutrition management*, *chronic disease and women’s health management*, *children’s skin health and vision issues*, *nutritional science and food controversies*, *cancer prevention and vaccine immunization*, *weight control and metabolic health*, and *environmental hazards affecting reproductive health*.

Further analysis of the online health information that was misjudged by all 4 models revealed a total of 2.44% (66/2708) of instances that were incorrectly classified by all 4 LLMs. The distribution of these misjudged instances across the 7 topics is shown in Table 8. While some topics had a higher overall proportion in the dataset, the misjudgment rates varied. The *food and maternal-infant nutrition management* topic had the most misjudged instances (18/548, 3.3%), whereas *environmental hazards affecting reproductive health* had the least (4/230, 1.7%). However, in terms of misjudgment ratios within each topic, the highest misjudgment rate was observed in *food and maternal-infant nutrition management* at 3.3% (18/548), followed by *nutritional science and food controversies*, with a misjudgment rate of 2.9% (11/374).

**Table 8:** Distribution and proportions of topics and misjudged information within the dataset (N=2708).

Topic number	Topic name	Total sample size, n (%)	Misjudged sample size relative to the topic’s total, n/N (%)
2	Food and maternal-infant nutrition management	548 (20.2)	18/548 (3.3)
4	Chronic disease and women’s health management	469 (17.3)	7/469 (1.5)
0	Children’s skin health and vision issues	433 (16)	12/433 (2.8)
1	Nutritional science and food controversies	374 (13.8)	11/374 (2.9)
6	Cancer prevention and vaccine immunization	363 (13.4)	8/363 (2.2)
5	Weight control and metabolic health	291 (10.7)	6/291 (2.1)
3	Environmental hazards affecting reproductive health	230 (8.5)	4/230 (1.7)

## Discussion

### Principal Findings

Within the task of online health information identification, this study evaluated the performance of 4 LLMs: ChatGPT-4, ChatGPT-3.5, iFLYTEK Spark, and Ernie Bot. Semantic similarity analysis was used to assess the similarity between the texts generated by these models and expert explanations, aiming to better understand the differences between the models. While these models are capable of performing this task in most cases, they cannot fully replace the judgment of human experts [34]. Specifically, health misinformation is prevalent in certain topics such as smoking products, opioids, and marijuana, with misinformation reaching 87% in some cases. Misinformation about vaccines, especially the human papillomavirus vaccine, and noncommunicable diseases such as cancer also remains common, with rates of 43% and 40%, respectively [35].

ChatGPT can be classified into ChatGPT-3.5 and ChatGPT-4, which exhibit differences in their performance in online health information identification tasks. ChatGPT-4 demonstrates slight improvements in accuracy and precision, showcasing its enhanced ability to deliver detailed explanations for intricate health-related queries [36]. In contrast, while ChatGPT-3.5 possesses certain natural language processing capabilities and logical reasoning skills, the texts it generates are generally shorter. Research has shown that ChatGPT-4 outperforms ChatGPT-3.5 and Google Bard in terms of accuracy and self-correction ability when addressing myopia-related queries [37]. Therefore, this study suggests that ChatGPT-3.5 has a comparatively weaker performance in managing specific details and ensuring accuracy than ChatGPT-4. Furthermore, ChatGPT-4 has improved logical coherence, enabling it to better integrate professional terminology and provide more compelling explanations. By comparison, Ernie Bot and iFLYTEK Spark exhibit notable strengths in Chinese-language processing and cultural comprehension. These 2 models are likely to align more closely with the thinking patterns and cultural contexts of Chinese users when generating explanatory texts. In particular, Ernie Bot has the potential to provide relatively personalized health recommendations based on user needs and contextual information. In summary, the 4 LLMs exhibit distinct characteristics in the context of online health information identification. Each model provides varying degrees of explanatory text support, assisting users in evaluating the reliability of online health information.

Semantic similarity analysis revealed that, although the overall similarity was relatively high, there were notable variations between the models in specific cases. For instance, ChatGPT-3.5 recorded the lowest semantic similarity score of 0.009, markedly lower than those of the other models, whereas Ernie Bot demonstrated a comparatively higher minimum similarity score of 0.0874, reflecting greater stability. This discrepancy may be attributed to the diverse strategies used by these models in processing online health information. Furthermore, an analysis of the coefficient of variation revealed the consistency of semantic similarity across different models. ChatGPT-4, ChatGPT-3.5, Ernie Bot, and iFLYTEK Spark exhibited coefficients of variation of 0.0856, 0.0922, 0.0859, and 0.0866, respectively. Lower coefficients of variation indicate greater stability and consistency in the semantic similarity between the model-generated and expert-generated texts. From this perspective, ChatGPT-4 and Ernie Bot exhibited greater semantic stability. Analysis of the content revealed that model-generated explanatory texts differed from expert-generated texts in aspects such as focus and comprehensiveness. Expert explanations tended to emphasize the application of medical knowledge and risk evaluation, providing in-depth analysis while frequently incorporating professional terminology and clinical context. In contrast, while the model-generated texts were more accessible and convey information succinctly, they often lacked depth and contextual support, which may lead to omissions of key information.

When addressing specific professional domains or emerging health issues, AI models may exhibit misunderstandings or provide insufficient information, which is related to issues of data availability, quality, and privacy [38]. For example, on COVID-19–related claims such as the following—“COVID-19 is a self-limiting disease”—judgments require up-to-date research and data [39]. However, models may struggle to make accurate assessments due to outdated training data. The KFF Health Misinformation Tracking Pilot Poll indicates that a significant proportion of the US population is exposed to and holds inaccurate beliefs about topics such as COVID-19, gun violence, and reproductive health. This underscores the urgent need for more reliable information sources and greater trust in health media. In light of this, both the general public and LLMs are susceptible to misinformation on complex and evolving health issues such as COVID-19 [40]. Given the challenges that both face in accessing accurate and up-to-date information, it is increasingly crucial to verify health-related content through trusted expert sources. Similarly, for emerging health concerns such as “Vitamin D supplementation can prevent uterine fibroids” or “Children wearing N95 masks may suffer irreversible harm,” models are prone to misjudgments. These issues often lack sufficient research or scientific consensus, complicating accurate identification. In addition, discussions surrounding nutritional products (eg, “Fish oil can protect the heart”) and emerging health technologies (eg, “Laser surgery can cure myopia”) may result in inaccuracies due to the models’ limited medical knowledge. Therefore, users are advised to exercise caution and cross-verify model-generated health information with other reliable sources.

When analyzing online health information in which LLM and expert judgments disagree, certain recurring patterns and potential causes were identified. First, in cases in which experts deemed information false, model misjudgments often stemmed from their adoption of popular yet scientifically unverified health concepts. This highlights the challenges that models face in distinguishing verified medical knowledge from unsubstantiated popular beliefs during training, particularly when evaluating vague or exaggerated claims. Such ambiguity in these claims not only increases the difficulty of model interpretation but also reveals their limitations in recognizing accurate information. Second, when models misjudged expert-verified true information as false, it reflected the limitations they encounter in handling complex biomedical knowledge. Insufficient training data and limitations in knowledge bases may hinder the models’ ability to accurately recognize information specific to professional domains. In addition, their limited ability to understand and apply context often results in higher error rates for context-specific analysis. Collectively, these findings reveal the models’ shortcomings in handling popular information, professional knowledge, and context-dependent data [41]. This provides a direction for future improvements in model training methods and optimization strategies, particularly in enhancing their ability to understand scientific knowledge and contextual information.

In this study, all 4 LLMs misjudged 66 online health information samples, with none providing correct classifications. Although these misjudgments accounted for only 2.44% (66/2708) of the dataset, their potential implications in medical informatics applications should not be overlooked. Misjudged online health information may contribute to suboptimal health decisions. For instance, misjudgments in the *food and maternal-infant nutrition management* domain may lead to inappropriate dietary choices during pregnancy, potentially affecting both maternal and infant health. Similarly, misjudgments in *nutritional science and food controversies* may cause individuals to disregard evidence-based dietary recommendations, increasing the risk of chronic diseases. In collaborative AI-driven environments, such errors may propagate across models, amplifying their impact. Misjudgments in critical areas such as *cancer prevention and vaccine immunization* could lead to confusion or vaccine hesitancy, thereby undermining public health initiatives. At the system level, misjudged information in *chronic disease management* *and* *women’s health management* could influence health care policies, potentially resulting in suboptimal health interventions and delays in patient care. On a broader scale, misjudged health data could disrupt international public health efforts, particularly during global health crises, by influencing policy decisions and international collaborations. Despite the demonstrated accuracy of these models, minimizing misjudgments remains a priority. Future research should prioritize model optimization incorporating expert feedback and multi-tier validation to enhance reliability in health information processing.

### Limitations and Future Work

While this study conducted a comprehensive analysis of LLMs in online health information identification, several limitations remain to be addressed. First, the quality and diversity of the training data may constrain the models’ comprehension of domain-specific medical knowledge, particularly in handling recent advancements and intricate biomedical concepts. Although trained on large-scale corpora, these models are not specifically optimized for the health information domain, nor do they comprehensively cover the latest medical advancements and complex biomedical concepts. As a result, the models may exhibit errors when handling cutting-edge research or highly specialized medical content. Second, this study primarily drew on data made publicly available by rumor-debunking websites. However, these sources may not encompass the full spectrum of health-related information, particularly content originating from clinical settings or platforms with limited access. As most of the data were gathered from Chinese websites, there is a possibility of regional or systemic bias in how information is categorized as *true* or *false*. The reliance on a specific dataset raises concerns about the generalizability and external validity of the research findings. This issue is even more important when LLMs are expected to handle false health information in global or culturally diverse settings. As a result, the experimental dataset may not fully represent real-world clinical information, which could lead to differences in the models’ performance in practical applications.

In addition, this study primarily evaluated the effectiveness of LLMs in identifying true and false health information; however, it did not fully account for the diversity of user interactions, particularly the influence of health literacy on query formulation. Recent research has shown that patients and consumers differ significantly in their access to digital technology, ability to assess information accuracy, and trust in digital health tools, all of which affect their interactions with AI-based health information systems [42]. It is worth noting that lower health literacy not only hinders communication in health care settings but also impacts individuals’ ability to search for and comprehend health information in online environments [43]. In real-world applications, users with varying health literacy levels may interact with AI models differently. Individuals with higher health literacy are likely to pose queries with appropriate medical terminology and more precise expressions, whereas those with lower health literacy may struggle to accurately formulate questions or may use simplified language. This disparity in query formulation can affect how models interpret and respond to health information, thereby impacting the effectiveness and practicality of the models. For individuals with lower health literacy, model outputs may be too complex, lack necessary context, or fail to provide adequate clarification, leading to misunderstandings or an inability to access meaningful information. Therefore, future research should explore how to optimize LLMs to better accommodate users with different levels of health literacy. Specifically, models could assess the complexity of a user’s query (such as the use of medical terms) and adjust the response accordingly. For users with lower health literacy, models could provide simplified language and clearer explanations, whereas for users with higher health literacy, more detailed and professional responses could be offered. In addition, providing multiple versions of answers based on users’ comprehension levels could enhance user experience and improve the overall effectiveness of health information retrieval through AI models.

Furthermore, this study primarily evaluated semantic similarity between model-generated texts and expert explanations. While this approach provides an objective measure of output quality, it may overlook critical nuances in online health information, such as contextual dependencies and the precision of medical terminology. Semantic similarity metrics often rely on general text comprehension, potentially resulting in a 1D assessment. Future research should incorporate more comprehensive evaluation frameworks that account for the complexity of medical contexts. Finally, while multiple pretrained language models were compared, the evaluation was limited to their default architectures and parameter settings without further exploration or optimization of the models’ structures. In future research, we will explore fine-tuning models using domain-specific databases and developing customized architectures to enhance their capability in identifying and interpreting online health information.

### Conclusions

On the basis of the results of this study, we found that the performance of the 4 LLMs in identifying online health information varied. Specifically, ChatGPT-4 demonstrated higher accuracy, with both accuracy and recall rates outperforming those of the other models, indicating strong abilities in understanding and assessing online health information. The κ coefficient analysis also indicated that ChatGPT-4 had the highest level of consistency with the ratings provided by the research team members, further confirming its superiority in credibility assessment. Notably, Ernie Bot showed a higher similarity to expert explanatory texts in both semantic similarity and text length analyses, indicating that Ernie Bot may hold greater potential in Chinese contexts, particularly in handling complex or specialized online health information. In addition, in the analysis of misjudgments by the 4 models, 2.44% (66/2708) of the information did not align with expert judgment. The misjudgment rates were highest within the themes of *food and maternal-infant nutrition management* and *nutritional science and food controversies*. These findings suggest that future research should focus on these areas to enhance the models’ performance in identifying online health information, improving the accuracy of their judgments. Although this study provides valuable insights into the evaluation of model efficacy, it has certain limitations, such as the potential impact of sample selection on the generalizability of the results and the lack of consideration for the models’ handling of different types of online health information. Therefore, future research should expand the sample size to include a broader range of health topics and explore the applicability of the models in real-time data processing across diverse information sources. In summary, this study offers empirical support for the use of LLMs in online health information identification, highlighting their potential to improve public health literacy. As technology continues to advance, further exploration and refinement of these models’ applications will be an important focus for future research, aiming for more accurate online health information identification and promoting more effective public health interventions.

## Appendix

Multimedia Appendix 1 Debunking platforms and their websites.

Multimedia Appendix 2 Partial identification results of pretrained language models.

Multimedia Appendix 3 Examples with high semantic similarity between the large language models and the expert explanations.

Multimedia Appendix 4 Examples with low semantic similarity between the large language models and the expert explanations.

## Abbreviations

AI: artificial intelligence
API: application programming interface
FP: false positive
GRADE: Grading of Recommendations Assessment, Development, and Evaluation
LDA: latent Dirichlet allocation
LLM: large language model
PCA: principal component analysis
RCT: randomized controlled trial
TP: true positive
USMLE: US Medical Licensing Examination

## Data Availability

The datasets generated or analyzed during this study are available from the corresponding author on reasonable request.
