# Supplementary material for: Identification of Online Health Information Using Large Pretrained Language Models: Mixed Methods Study
Source: J Med Internet Res. 2025 May 14;27:e70733. doi: 10.2196/70733 (PMC12120363; doi:10.2196/70733)
Supplement: Multimedia Appendix 1 [file jmir_v27i1e70733_app1.docx]

**Attachment**

| **Debunking Platform** | **Verification Sources** | **Corresponding URLs** |
| --- | --- | --- |
| Tencent FactCheck  (https://jiaozhen.qq.com) | World Health Organization (WHO) | [https://www.who.int](https://www.who.int/) |
|  | National Medical Products Administration (NMPA) | [https://www.nmpa.gov.cn](https://www.nmpa.gov.cn/) |
|  | Xinhua News Agency (Official Website) | [http://www.xinhuanet.com](http://www.xinhuanet.com/) |
|  | CCTV News (Official Website) | [https://news.cctv.com](https://news.cctv.com/) |
|  | People's Daily (Official Website) | [http://www.people.com.cn](http://www.people.com.cn/) |
|  | People's Daily - Life Times (Official Website) | [http://www.lifeweek.com.cn (Part of People's Daily)](http://www.lifeweek.com.cn/) |
