# Supplementary material for: Identification of Online Health Information Using Large Pretrained Language Models: Mixed Methods Study
Source: J Med Internet Res. 2025 May 14;27:e70733. doi: 10.2196/70733 (PMC12120363; doi:10.2196/70733)
Supplement: Multimedia Appendix 2 [file jmir_v27i1e70733_app2.docx]

**Partial identification results of pretrained language models**

| Claim | ChatGPT-4 (OpenAI) | ChatGPT-3.5 (OpenAI) | iFLYTEK Spark | Ernie Bot (Baidu) |
| --- | --- | --- | --- | --- |
| “Diabetes is caused by eating too much sugar” | “The main cause of diabetes is the inability of pancreatic ß-cells to secrete insulin properly, or the body’s tissue cells becoming insulin resistant, leading to high blood sugar. Although eating too much sugar may increase the risk of diabetes, it is not the direct cause.” | “Diabetes is a chronic metabolic disease primarily caused by insufficient insulin secretion or impaired insulin response in cells. While excessive sugar intake may increase the risk of diabetes, the onset and progression of diabetes are influenced by multiple factors and cannot be simply attributed to eating too much sugar. Therefore, diabetes is not solely caused by eating too much sugar.” | “Diabetes is related to various factors, including genetics, environment, and lifestyle. While excessive sugar intake may increase the risk of the disease, it should not be simply concluded that diabetes is caused by eating too much sugar. The pathogenesis of diabetes is complex, involving insulin secretion and action abnormalities. Therefore, diabetes should not be simply attributed to eating too much sugar.” | “The cause of diabetes is not just eating too much sugar. While a high-sugar diet may increase the risk of diabetes, the real cause of diabetes usually involves multiple factors, including genetics, lifestyle, obesity, and lack of exercise. Additionally, diabetes is categorized into different types, such as Type 1 and Type 2 diabetes, which have different causes and treatments. Therefore, attributing diabetes solely to eating too much sugar is inaccurate.” |
| “Rice washing water can improve skin” | “Rice washing water contains some vitamins and minerals, which have certain moisturizing and whitening effects on the skin. Therefore, washing or applying rice washing water to the face may have certain cosmetic benefits. However, since each person’s skin type and condition vary, the effectiveness of rice washing water in beauty care may differ from person to person.” | “Rice washing water contains vitamins B and C, as well as minerals, making it a natural skincare product. Regular use of rice washing water for cleansing can help alleviate skin dryness, brighten the complexion, and provide other cosmetic benefits.” | “Rice washing water contains a large amount of vitamins B and minerals, which have cosmetic and skincare benefits. It can be used to wash the face or soak feet, contributing to skin whitening and smoothness. However, it is important to note that rice washing water should ideally be freshly made and not stored for too long, as bacteria may grow, which could be harmful to the skin.” | “Rice washing water contains starch, vitamins, and minerals, which have certain moisturizing and whitening effects on the skin. However, the effectiveness may vary depending on individual differences, and rice washing water should not be solely relied upon for skincare.” |
| “Men can also have breast diseases.” | “Although men are less likely to develop breast diseases, they do have breast tissue and are therefore at risk of conditions such as mastitis, fibrocystic breast changes, and breast cancer. While the likelihood of men developing breast diseases is lower, it is not impossible. Therefore, men should also pay attention to their breast health and regularly perform self-examinations and medical check-ups. ” | “Although men have less breast tissue than women, they can still develop breast conditions, such as mastopathy and mastitis. Therefore, men should also pay attention to breast health, regularly perform self-exams, and seek medical attention promptly if any abnormalities are detected. ” | “Men also have breast tissue, so theoretically, they can develop breast conditions. Although this is relatively rare, it is not impossible. For example, male breast cancer is a real disease, although its incidence is much lower than in women. ” | “Although men are relatively less likely to develop breast conditions, they also have breast tissue, so they can still develop conditions such as mastitis, fibrocystic breast changes, or breast cancer. While the likelihood of men having breast conditions is lower, it is not impossible. Therefore, men should also pay attention to their breast health, regularly perform self-exams, and have check-ups. ” |
