# Supplementary material for: Identification of Online Health Information Using Large Pretrained Language Models: Mixed Methods Study
Source: J Med Internet Res. 2025 May 14;27:e70733. doi: 10.2196/70733 (PMC12120363; doi:10.2196/70733)
Supplement: Multimedia Appendix 3 [file jmir_v27i1e70733_app3.docx]

**Examples with high semantic similarity between the large language models and the expert explanations**

| Model | Similarity score | Text content | Expert or institution explanatory text | Model-generated comment |
| --- | --- | --- | --- | --- |
| ChatGPT-3.5 (OpenAI) | 0.9686 | Sugar increases the risk of cancer (false). | “A lump or nodule is not necessarily cancer; it can be non-tumorous (such as an inflammatory mass) or a benign tumor. However, it should not be taken lightly, and further examination is recommended for a definitive diagnosis.” | “A lump or nodule is not necessarily cancer; it could be caused by non-malignant conditions such as inflammation or cysts, but it could also be a malignant tumor. Therefore, the appearance of a lump or nodule requires prompt medical attention for further examination to determine the cause.” |
| iFLYTEK Spark | 0.9658 | Cosmetic allergies easily lead to vitiligo (false). | “Vitiligo is a pigment disorder, and its exact cause remains unclear. It is currently believed that vitiligo has a genetic predisposition and results from a combination of internal and external factors. Cosmetic allergies are a type of allergic skin condition and are different from vitiligo. There is no definitive connection between cosmetic allergies and vitiligo, making the statement ‘cosmetic allergies easily lead to vitiligo’ scientifically unfounded. However, cosmetics can potentially trigger vitiligo, but the likelihood is very low, and it can easily be confused with chemical leukoderma.” | “Cosmetic allergies and vitiligo are two distinct conditions with different causes and mechanisms. Cosmetic allergies are primarily due to the skin’s reaction to certain ingredients in cosmetic products, while vitiligo is a pigment loss disorder with an unclear cause, potentially related to genetic, immune, neurological, and environmental factors. Therefore, cosmetic allergies do not cause vitiligo.” |
| Ernie Bot (Baidu) | 0.9498 | The calcium supplementation effect of kidney beans is stronger than that of milk (false). | “Kidney beans are rich in dietary calcium, and their calcium content per unit weight even exceeds that of milk. However, according to the Dietary Guidelines for Chinese Residents, the recommended intake of legumes for children is much lower than that of dairy products. Therefore, when considering calcium intake over a specific period, kidney beans provide less calcium than milk. The bioavailability of calcium in kidney beans is lower than that in milk, as the calcium in milk and the effects of lactic acid fermentation make it more efficient for calcium absorption. So, in terms of calcium absorption, kidney beans are inferior to milk. That being said, relying solely on milk for calcium supplementation is insufficient; it is also necessary to obtain calcium from other sources such as algae, soy products, and shrimp.” | “Although kidney beans do contain some calcium, it is not accurate to say that their calcium supplementation effect is stronger than that of milk. Milk is a high-quality calcium source with both high calcium content and absorption rate. In contrast, while kidney beans also contain calcium, their calcium content is relatively lower, and factors such as cooking methods may affect the absorption rate of calcium from kidney beans, which may not be as efficient as milk. Therefore, from a calcium supplementation perspective, milk remains a more ideal choice.” |
| ChatGPT-4 (OpenAI) | 0.9705 | Myopia, hyperopia, and astigmatism are all refractive errors (true). | “Myopia, hyperopia, and astigmatism are all types of refractive errors. Myopia occurs when the curvature of the cornea and lens is too steep, or the eyeball is too long, causing light to focus in front of the retina. Hyperopia happens when the curvature of the cornea and lens is too flat, or the eyeball is too short, causing light to focus behind the retina. Astigmatism occurs when the shape of the eyeball is irregular, causing light to refract in such a way that it does not form a single focal point on the retina.” | “Myopia, hyperopia, and astigmatism are all abnormalities of the refractive state, known as refractive errors. Myopia occurs due to an excessively long eyeball axis or an overly strong refractive system, causing distant objects to focus in front of the retina. Hyperopia happens when the eyeball axis is too short or the refractive system is too weak, resulting in distant objects being focused behind the retina. Astigmatism occurs when the curvature of the cornea or lens is uneven, causing light entering the eye to fail to focus uniformly, leading to blurred vision. Therefore, myopia, hyperopia, and astigmatism all fall under the category of refractive errors.” |
