# Supplementary material for: Identification of Online Health Information Using Large Pretrained Language Models: Mixed Methods Study
Source: J Med Internet Res. 2025 May 14;27:e70733. doi: 10.2196/70733 (PMC12120363; doi:10.2196/70733)
Supplement: Multimedia Appendix 4 [file jmir_v27i1e70733_app4.docx]

**Examples with low semantic similarity between the large language models and the expert explanations**

| Model | Similarity score | Text content | Expert text | Model-generated comment |
| --- | --- | --- | --- | --- |
| ChatGPT-3.5 (OpenAI) | 0.3684 | Taking vitamin C can boost immunity and prevent colds (false). | “Supplementing with extra vitamin C cannot prevent colds; this is similar to taking dietary supplements. The only effective methods for preventing colds include regular exercise, adequate sleep, avoiding overexertion and exposure to cold, frequent handwashing, maintaining a clean and well-ventilated home environment, limiting activities in crowded places, avoiding contact with individuals with respiratory infections, and getting vaccinated against the flu.” | “Vitamin C can contribute to boosting the immune system and may help in preventing colds to some extent. However, it cannot completely prevent colds. It is still important to maintain a balanced diet and healthy lifestyle habits.” |
| iFLYTEK Spark | 0.3980 | Applying a face mask can hydrate the skin (false). | “The skin has limited water absorption; only a small amount of water is absorbed by the intact epidermis. When the external humidity is significantly higher than the moisture content within the epidermis, the skin can absorb some water, but after about ten minutes, the skin stops absorbing and becomes saturated. This is why there is no need to leave a face mask on for more than 10 minutes. After removing the mask, the small amount of water absorbed will evaporate. Face masks are not useless, but their benefits can be summarized into two categories: promoting the absorption of active ingredients and providing physical cooling. Furthermore, ‘hydration’ is a misleading concept. In fact, all methods that truly improve epidermal hydration ultimately work by reducing water loss through the skin, which should be called ‘moisturization.’ The main product for daily moisturizing is a moisturizer. Lastly, wet compresses can damage the skin’s lipid layer, and frequent wet compresses are harmful to the skin barrier. Therefore, the use of face masks should be limited to 2-3 times per week and should not be excessive.” | “Face masks are a common skincare product, and one of their main functions is to provide moisture and nutrients to the skin. The various ingredients found in face masks, such as hyaluronic acid, glycerin, etc., have excellent moisturizing effects and can effectively replenish the skin with moisture. Of course, the hydration effect may vary between different face mask products, and the skin’s ability to absorb these ingredients also differs, so the results after using a face mask can vary from person to person.” |
| Ernie Bot (Baidu) | 0.2769 | Taking vitamin C can boost the immune system and help prevent colds (false). | “Supplementing with extra vitamin C cannot prevent colds, just like taking dietary supplements. The real ways to prevent colds are regular exercise, getting enough sleep, avoiding overexertion, staying warm, washing hands frequently, keeping the home environment clean and well-ventilated, minimizing visits to crowded places, avoiding contact with individuals with respiratory infections, and getting the flu vaccine to prevent the flu.” | “Vitamin C plays an important role in the human immune system and helps enhance the body’s resistance to diseases. Some studies suggest that adequate supplementation of vitamin C may help prevent common illnesses like colds. However, for the general population, a balanced diet can typically meet the body’s vitamin C requirements, and excessive intake of vitamin C may lead to side effects. Therefore, it is advisable to consult a doctor or nutritionist before taking vitamin C supplements.” |
| ChatGPT-4 (OpenAI) | 0.4545 | Shampoo opens the hair cuticle, whereas conditioner closes it (false). | “The hair cuticle is made up of the outermost layer of dead cells, also known as the cuticle scales. They function like fish scales, protecting the inner structure of the hair. When you wash your hair, it absorbs water and swells, causing the cuticle scales to lift (the cuticle scales only lift, not open). The lifting of the cuticle scales does not mean that the ‘nutrients’ in the shampoo can be absorbed by the hair, as these chemicals cannot penetrate the hair structure. After washing, the cuticle scales do not return to their original position, so they no longer provide protection to the hair. Conditioners contain moisturizing ingredients that, while they cannot close the lifted cuticle scales, can form a lubricating layer around them, helping to smooth and protect the hair.” | “To some extent, this statement is scientifically grounded. Shampoos contain surfactants, which help remove oils and dirt from the hair. This process can cause the outer layer of the hair, the cuticle scales, to lift. On the other hand, conditioners contain softening agents and oils, which help close the lifted cuticle scales, making the hair appear smoother and softer. Therefore, this claim is reasonable based on the fundamental principles of hair cleaning and care.” |
